# Addressing Bureaucratic Burdens on the Portuguese National Health Service: A Simplification Experience Aiming for Value-Based Healthcare

**DOI:** 10.3390/healthcare13070821

**Published:** 2025-04-04

**Authors:** Francisco Goiana-da-Silva, Raisa Guedes, Filipa Malcata, Juliana Sá, Miguel Cabral, Rafael Vasconcelos, Soraia Costa, Inês Morais-Vilaça, Lara Pinheiro-Guedes, João Sarmento, Filipe Costa, Rita Moreira, Fátima Fonseca, Jaime Alves, Marisa Miraldo, Alexandre Morais Nunes, Hutan Ashrafian, Ara Darzi, Fernando Araújo

**Affiliations:** 1 Centre for Health Policy, Institute of Global Health Innovation, Imperial College London, London W2 1NY, UK; 2 Nova Medical School, Travessa de Estêvão Pinto—Campus de Campolide, Universidade Nova de Lisboa, 1099-085 Lisbon, Portugal; 3 Faculdade de Ciências da Saúde, Universidade da Beira Interior, 6200-506 Covilhã, Portugal; 4 Unidade Local de Saúde São João, 4200-319 Porto, Portugal; 5 Unidade Local De Saúde Santo António, 4099-001 Porto, Portugal; 6 Unidade Local De Saúde Região de Leiria, 2410-197 Leiria, Portugal; 7 Unidade Local De Saúde Póvoa de Varzim/Vila do Conde, 4490-421 Póvoa de Varzim, Portugal; 8 Unidade Local De Saúde Gaia/Espinho, 4434-502 Vila Nova de Gaia, Portugal; 9 Unidade Local De Saúde Tâmega e Sousa, 4560-136 Penafiel, Portugal; 10 Public Health Research Center, Nova National School of Public Health, Universidade Nova de Lisboa, 1600-560 Lisbon, Portugal; 11 Nova School of Business and Economics, Travessa de Estêvão Pinto—Campus de Campolide, Universidade Nova de Lisboa, 1099-085 Lisbon, Portugal; 12 Unidade Local De Saúde Região de Aveiro, 3814-501 Aveiro, Portugal; 13 Ministério das Finanças, Governo de Portugal, 1149-009 Lisbon, Portugal; 14 Department of Economics and Public Policy, Centre for Health Economics and Policy Innovation Imperial College Business School, South Kensington Campus, Imperial College London, London SW7 2AZ, UK; 15 Centre for Public Administration and Public Policies, Institute of Social and Political Sciences, University of Lisbon, 1300-663 Lisbon, Portugal; 16 Department of Surgery and Cancer, Faculty of Medicine, Imperial College London, London W12 0NN, UK; 17 Faculty of Medicine, University of Porto, 4200-319 Porto, Portugal

**Keywords:** healthcare management, innovation, outcomes, bureaucracy, Portuguese NHS, healthcare reform, value-based healthcare

## Abstract

Background/Objectives: The Portuguese NHS has embarked on an administrative restructuring aimed at enabling healthcare professionals, particularly family doctors, to focus on direct patient care and improve overall healthcare outcomes. This article details these measures, their initial benefits for patients and professionals, and explores future strategies to further integrate levels of care and leverage technology to enhance efficiency, patient-centeredness, and ultimately, population health. Methods: Each measure was evaluated to estimate its potential impact on the four pillars of the value-based healthcare (VBHC) framework. Results: We found that most measures aimed at reducing bureaucracy had an estimated impact on more than two of the four pillars. Conclusions: Thus, we conclude that the reduction in bureaucracy will tend to address several of the pillars of the VBHC framework and should be considered as a steppingstone in the process of increasing VBHC.

## 1. Introduction

A minimal level of formalized processes is vital in complex systems, especially in healthcare, where rules and processes ensure quality, efficiency, and safety. However, excessive bureaucracy can hinder healthcare activities, reducing accessibility to care and preventing staff from prioritizing patient needs, effectively managing risk, empowering patients and colleagues, and adapting to changing circumstances [1]. Additionally, this can adversely affect professionals’ well-being, morale, and retention [2]. “Eliminating Time-Consuming Documentation” was a problem identified by Sinsky et al. when pursuing joy in primary care practice [2]. An administrative burden refers to an individual’s experience of policy implementation as burdensome, typically categorized into three main types of costs: learning, psychological, and compliance costs. Learning costs involve the effort to gather and understand information about public services. Psychological costs include the stigma, loss of autonomy, and stress associated with navigating administrative processes. Compliance costs refer to the effort required to adhere to administrative rules and requirements [3]. In this context, documentation and bureaucratic tasks are considered part of the overall administrative burden.

Bureaucratic dimensions divert healthcare professionals from their primary focus: to deliver the best outcomes for patients. Instead of dedicating time to focus on and attend to their patients’ needs, healthcare providers are compelled to concentrate on processes, documentation, and obtaining various authorizations [3]. In the absence of appropriate training and incentives to perform these tasks, this bureaucratic burden leads to increased dissatisfaction and burnout among healthcare professionals and a decline in the overall quality of medical care, as the emphasis on administrative tasks reduces the time available for direct patient care [4,5,6], thus creating a management burden fixed only with innovative ideas [7].

### 1.1. Bureaucracy and Burden

The administrative burden in healthcare is a significant issue affecting systems worldwide. This burden encompasses the extensive tasks required to comply with regulations, manage finances, handle patient data, and ensure adherence to health laws and standards. These tasks can divert resources from patient care and increase operational complexity.

One key area of burden is regulatory compliance. Healthcare providers must follow numerous regulations related to healthcare delivery, safety standards, patient privacy, among others. Ensuring compliance requires substantial documentation and constant monitoring, consuming both time and resources [7]. Additionally, documentation requirements—such as maintaining comprehensive medical records for continuity of care, legal protection, and billing—further contribute to this. While electronic health record [EHR] systems aim to streamline this process, they also are often time consuming due to complex interfaces [8].

Another challenge is quality assurance and reporting. Healthcare providers must continually collect, analyze, and submit data to demonstrate compliance with regulatory standards. This quality oversight, while necessary, adds to the administrative load [9]. The financial impact is also considerable, as administrative tasks in European healthcare systems represent a significant portion of expenditures. Managing paperwork, billing, compliance, and technology systems can drive up costs and detract from care efficiency [10]. Moreover, administrative complexities increase operational costs and can lead to inefficiencies and delays in treatment, sometimes compromising patient care [11]. Furthermore, excessive bureaucracy contributes to physician burnout, with clinicians spending more time on paperwork than patient care, which affects job satisfaction and the quality of care provided [12].

To address these issues, multiple avenues are being pursued to reduce administrative burdens in healthcare. Regulatory reform is one approach, aiming to simplify and streamline healthcare regulations to reduce unnecessary complexity and allow providers to focus more on patient care [13]. Technology solutions are also essential, as more intuitive and integrated EHR systems and automation can alleviate the administrative burden on healthcare staff [14]. Additionally, process improvement strategies like lean management help organizations become more efficient and patient centered, reducing redundant processes and improving workflow [15].

Reducing the administrative burden is crucial for improving healthcare delivery, lowering costs, and enhancing satisfaction for both patients and providers. A shift toward patient-centered care, where the focus is on outcomes that matter to patients, can promote more effective and personalized treatments [16]. This, in turn, can lead to the efficient use of resources by discouraging unnecessary procedures, stimulating innovation in medical technologies and pharmaceuticals [17], and ultimately increasing the health outcomes achieved per dollar spent by prioritizing preventative care and effective interventions [18]. In Table 1, a summary of bureaucratic burden impacts and their conceptual framework can be seen for an easier comprehension of the problems faced and possible solutions.

### 1.2. The Need to Address Bureaucracy in Value-Based Healthcare

In recent years, many countries have undertaken measures to streamline bureaucratic processes, aligning with efforts to achieve the United Nations [UN] Sustainable Development Goals targets [19]. The COVID-19 pandemic accelerated this trend, prompting a reassessment of procedures due to pandemic mitigation restrictions on mobility and contact rates. The United Kingdom [UK] launched the program “Busting bureaucracy: empowering frontline staff by reducing excess bureaucracy in the health and care system in England” in 2020 [1] and is using its new integrated care system to rethink bureaucratic snares and make improvements from within [20]. Swedish care organizations are implementing planned changes and lean management, a philosophy that promotes streamlining processes [21]. In the United States of America [USA], initiatives like Patients Over Paperwork by the Centers for Medicare and Medicaid Services and efforts to increase healthcare workflow automation by the Office of the National Coordinator for Health Information Technology are underway [22,23]. Similarly, in Germany, there has been recognition of the significant time physicians spend on bureaucracy, highlighting the potential for digital solutions to mitigate this challenge [24].

These efforts to streamline healthcare bureaucracy are critical in addressing the evolving needs of healthcare systems worldwide. As organizations seek to improve efficiency, the focus is shifting towards optimizing resources and enhancing care delivery.

Worldwide, healthcare systems are under pressure to optimize the use of limited resources as they face rising costs associated with patients having multiple chronic conditions, changing clinical practices, and rapidly emerging technologies. In this context, the notion of ’value-based healthcare’ [VBHC], brought by Porter almost 20 years ago [25], is being increasingly utilized. ’Value’ is often perceived as ’health outcomes relative to monetized inputs’, but it encompasses more, considering the context and subjectivity involved in its definition and reality on its application [25,26].

The strategic goals of VBHC involve shifting from volume-based to value-based care models, which is a fundamental change in how healthcare success is measured [27]. For the Expert Panel on Effective Ways of Investing in Health nominated by the European Commission [25], the definition of healthcare value is built on four value pillars:

- Allocative Value [AV] is presented as an equitable distribution of resources across all patient groups.
- Technical Value [TV] is presented as an achievement of the best possible outcomes with available resources.
- Personal Value [PV] is presented as an appropriate care to achieve patients’ personal goals.
- Societal Value [SV] is presented as a contribution of healthcare to social participation and connectedness [25].

Bureaucracy in healthcare significantly impacts the core principles of VBHC by creating barriers and inefficiencies that hinder the equitable distribution of resources, achievement of optimal outcomes, provision of personalized care, and enhancement of social participation. In a model with economic limited resources, it is mandatory to allocate the best efficacy and efficiency with the investment produced along the chain of value produced. Firstly, these processes hinder the equitable distribution of resources across all patient groups, compromising fairness and access to healthcare. Secondly, these inefficiencies compromise the quality of outcomes that could be achieved [28], affecting the system’s ability to deliver high-quality care. Thirdly, bureaucratic complexities complicate the provision of personalized care tailored to individual patient goals, diminishing patient experiences. Lastly, these inefficiencies also detract from SV by making it more challenging for patients to receive timely and efficient care, affecting their ability to participate fully in society, and delivers a higher cost to achieve care and higher depreciation of the resources. Delays and inefficiencies in the healthcare system can lead to prolonged illness or disability, reducing individuals’ capacity to engage with their communities and contribute to societal well-being. According to Lorkowski et al., “Bureaucracy and healthcare are the terms that form a linguistic antithesis. In practice, it is paramount to balance the contradictions that are inherent in these concepts so that the bureaucratic procedures expected of medical staff would not dominate their work and would not deprive it of the primary role, which is the efficient and effective care over the sick” [29].

### 1.3. Portugal’s Bureaucratic Struggles in Healthcare

Portugal’s epidemiological and demographic profile before 2022 presented as a multifaceted array of challenges, spanning various domains of healthcare. Ageing and disease management of chronic conditions stand as prominent areas of concern, with the increase in non-communicable disease and multi-morbidity ailments posing significant burdens on healthcare resources [30]. Medication adherence, especially in older and chronic patients, has emerged as a critical issue, highlighting the importance of patient education and support mechanisms [31]. Health inequalities persist as a pressing issue, demanding targeted interventions to address socio-economic determinants of health and promote equitable access to healthcare services [32]. Related to these challenges, one of the primary issues of the Portuguese healthcare system has been the assignment of family doctors to patients. There is a substantial geographic variability in access to family doctors throughout the national territory [33]. Additionally, integration of care was hampered by the establishment of a complex plethora of organizations which reinforced a disconnection between the different settings of care [34].

Health workers are historically overworked, and administrative tasks can affect their efficiency. Studies show that physicians may spend 44.9% of their time on electronic health records [35]. In 2014, the Court of Auditors of Portugal issued an Audit Report evaluating the performance of Primary Care Units [36]. The report relied on a time analysis study focusing on the practices of family doctors in Portugal [37]. According to the findings, significant time was being devoted to administrative tasks such as communicating with other professionals about patients and addressing daily computer system malfunctions. Considering this, the court noted that this allocation of time could potentially be reduced to allow for additional patient consultations. Furthermore, the study emphasized the considerable time spent on prescription refills, as well as completing reports and certificates. Therefore, while Portugal is recognized by the World Health Organization’s Universal Health Coverage Service Coverage Index as a global reference for the coverage of essential health services [19,38], there are opportunities to streamline processes and enable frontline staff to allocate more of their time to patient needs.

Over the years, family doctors have been burdened with numerous administrative processes, including those related to the initial medical certification of sickness, subsequent renewals, and paperwork for incapacity, prescription renewals, and the renewal of medical procedures not performed due to patients’ time constraints and life problems. This also includes national program vaccine prescriptions and various medical certificates such as those for driving licenses, hunting permits, firearm licenses, and nautical sports certifications, among many others. A study from 2011 highlighted the multitude of tasks that family physicians perform in Portugal and found that the daily time spent by family physicians on tasks beyond consultations corresponds to 23% of their workload [39]. Another study, with the objective of identifying encounters with family doctors in the last decade in central Portugal, found that encounters classified with social problems as the reason recorded the highest growth dynamics from 2010 to 2018 [40], highlighting the need for better integration with Social Security.

Family doctors, however, were not the only ones overwhelmed. Many constraints also affected patients and other staff directly. Some of these inefficiencies included [i] recurrent and high-volume administrative tasks, like the scheduling of Primary Care Services [PCS] and supporting medical staff in paperwork management for diverse medical certificates and incapacity; [ii] non-digitalized data, as with surgery vouchers’ administration, as addressed below; and many others. These inefficiencies not only strained healthcare workers but also led to longer waiting times and decreased patient satisfaction. The lack of digital integration meant that support staff had to manually handle a vast array of paperwork, leading to errors and delays. Patients often had to make multiple visits to healthcare facilities for simple administrative processes, which could have been avoided with a more streamlined system. For example, a patient might need to visit their family doctor just to obtain a document related to a consultation with a different doctor at another facility.

**Figure 1:**
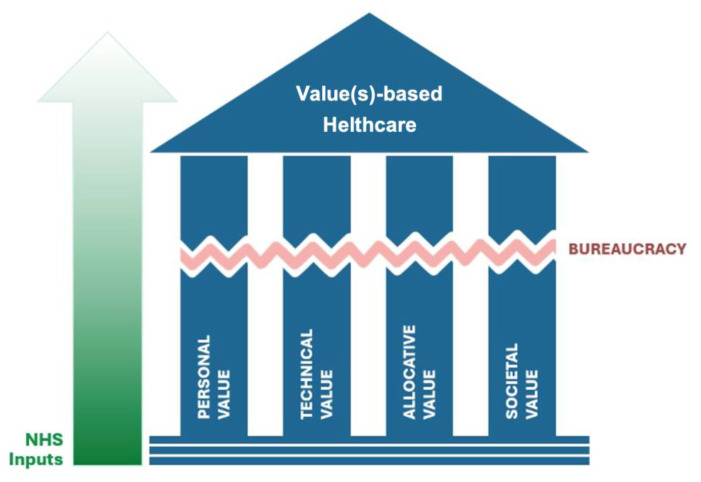
Framework depicting the impact of process simplification by the NHS executive board on the delivery of VBHC. Adapted from the Expert Panel on Effective Ways of Investing in Health from the European Commission [25].

The cumulative effect of these bureaucratic burdens was a significant reduction in the overall efficiency of the healthcare system. For example, surgery vouchers are documents that allow patients to schedule surgery at a hospital other than their primary one, including private hospitals, when waiting lists are excessive or recommended waiting times have been exceeded in their primary one. The manual processing of surgery vouchers not only delayed patient access to necessary surgeries but also increased the risk of losing or misplacing important documents. This inefficiency was mirrored in other areas, such as the management of primary care appointments, where a lack of coordinated scheduling systems could lead to missed appointments and underutilized time slots. This situation highlighted the urgent need for systemic reforms aimed at reducing bureaucratic hurdles through digitalization and better resource management. This would ensure that both patients and healthcare providers could operate within a more efficient and responsive healthcare system. In 2022, the Executive Board of the Portuguese National Health Service [EB-NHS] was established to simplify the National Health Service [NHS] organizational structure, previously characterized by numerous vertical layers and institutions. Since its inception, the EB-NHS has spearheaded several changes aimed at streamlining processes and reducing burdensome procedures that detract from patient care [41]. According to Gray, improvements in the quality, safety, and productivity of health services are not enough to meet the challenges that all health services face and will continue to face even if resources increase [26]. To address this, we must embrace VBHC alongside other models that mediate health outcomes at the correct cost to deliver value. This comprehensive understanding of ‘value’ offers a broader perspective than interpreting ‘value’ solely as monetary in the context of cost-effectiveness. Therefore, the above-mentioned framework was adapted by the EB-NHS to address the issue of bureaucracy and was used to systematize the process of change in Portugal during 2023 and 2024, through the leadership of the EB-NHS. Figure 1 illustrates a framework depicting the way the NHS executive board sees bureaucracy in the context of VBHC. Using as a basis the picture used by the Expert Panel on Effective Ways of Investing in Health from the European Commission on its report on VBHC [25], bureaucracy is depicted as a discontinuation of the pillars of VBHC, as if it is eroding them. When it is reduced, both the NHS’s efficiency in terms of the investments done and the delivery of VBHC improve.

Therefore, the purpose of this study is to systematize the predicted impact of seventeen priority measures identified by the EB-NHS to remove unnecessary bureaucratic burdens and increase workflow, using the VBHC framework above mentioned. In the case of twelve of these measures already implemented, the purpose is also to identify their initial impact and results.

## 2. Materials and Methods

This study is a qualitative analysis comparing the 2023/2024 measures proposed by the EB-NHS to reduce the bureaucratic burden with the framework of value-based healthcare proposed by the Expert Panel on Effective Ways of Investing in Health nominated by the European Commission. The study involves a thematic review of relevant scientific and policy literature, guided by the Standards for Reporting Qualitative Research [SRQR]. The study focuses on systemic changes and their impact on healthcare efficiency and outcomes, without involving human subjects or the collection of sensitive personal data.

### 2.1. Researcher Characteristics and Reflexivity

The research team for this study included members and advisors of the Portuguese NHS executive board, one of whom was the Chief Executive Officer of the EB-NHS. Additionally, the team included the Director of the Institute of Global Health Innovation at Imperial College London and the Chief Scientific Officer of Flagship Pioneering’s Preemptive Medicine and Health Security Initiative, along with other academic members not affiliated with the EB-NHS. This diverse composition allowed for maintaining reflexivity through discussion and challenging established assumptions.

### 2.2. Sampling Strategy

All measures were considered according to their initial implementation dates up to the point when data was collected, as indicated by the specific month and year for each measure. For predictions and estimates, initial data were used to extrapolate future numbers when present, or official governmental public estimates were considered.

### 2.3. Data Collection Methods, Instruments, and Technologies

When available, numerical data were collected directly from the informational systems of each measure, specifically official government data. On-site data collection was conducted using computers equipped with National Health Service [NHS]-approved software by health professionals present on site.

### 2.4. Data Analysis

A thematic analysis was applied to the obtained measures, that were categorized into sub-themes according to their main objectives. Each sub-theme was given a descriptive label conveying these objectives, in accordance with the VBHC concepts mentioned above [AV, TV, PV, SV]. As such, the VBHC concepts arose a priori to the analysis from a framework derived from the literature, as described above.

### 2.5. Data Trustworthiness

All Portuguese data points were sourced from official government data, accessed through the team members working at EB-SNS at the time. The team consisted of professionals with diverse specializations, enhancing the robustness and credibility of the data. Data dependability was ensured through rigorous documentation and accurate recording of all stages of the research process, providing a solid foundation for analysis.

### 2.6. Context of the Study

The study was conducted by healthcare providers and administrators involved in the implementation of reforms across various settings, including primary care and hospitals. The reforms being addressed encompass several areas, such as extending the duration of sick leave certification for specific clinical conditions, digitizing surgery vouchers, and integrating digital records for both public and private healthcare facilities. Additionally, measures like teleconsultation services through the Portuguese NHS Contact Centre [SNS24; a digital National health service in Portugal] platform and the use of AI-powered triage in dermatology were implemented in specialized care contexts. Each initiative was tailored to the unique needs of the respective clinical environments, addressing specific workflow bottlenecks and optimizing patient care delivery. It is also worth highlighting that some of the measures further developed the use of SNS24, which is an entity that started as a telehealth service via telephone line but has since been developing into other services such as an online portal and an app, among others.

## 3. Results

The key measures proposed by the EB-NHS, along with their corresponding sub-thematic analysis, are summarized in Table 2 and are detailed below.

### 3.1. Reducing Overly Complex Regulations and Work Redundancy

Self-certification of sickness up to three days, through contact with the Portuguese NHS Contact Center [SNS24]: Between 1 May 2023 and 21 April 2024, a total of 421,687 self-certification certificates for short-term sick leave were issued through SNS24. More than 99% of these self-certifications were completed via digital platforms, such as apps or the web portal, enabling fast and efficient monitoring of their issuance. The system covered 312,457 unique patients, while an additional 64,898 patients requested two certificates within the same year. This digital approach simplified the process of justifying short-term absences from work and significantly reduced the need for in-person visits to obtain the Certificate of Temporary Incapacity for Work [CIT], previously only issued by family doctors. With this measure in place, it is projected that the total number of certificates issued could reach 600,000 to 700,000 per year, representing the equivalent of 600,000 to 700,000 consultations that would otherwise be spent solely on documentation, thereby freeing up significant time for patient care [41].

Issuance of digital medical certificates of illness in emergency public services and private and social healthcare services: The issuance of digital medical certificates for sick leave has been expanded to include public emergency services, private, and social healthcare services. Previously, obtaining a medical declaration of illness required an appointment at a public primary healthcare facility with a family doctor. As of 1 March 2024, any healthcare service, including emergency and private/social healthcare facilities, is authorized to issue these digital certificates. Between 1 March and 21 April 2024, a total of 17,457 digital medical certificates for sick leave were issued through these services, representing approximately 4% of the total certificates issued during this period. Specifically, 5138 were issued in public emergency settings, 1737 in private emergency settings, and 10,582 in private or social healthcare organizations.

Extension of the prescription’s validity for medication and medical procedures up to 12 months: Before 1 April 2023, most prescriptions in the Portuguese healthcare system were valid for six months or less. With the legislation change championed by the EB-NHS and implemented on 1 April, the validity period for these prescriptions was extended to 12 months in a digital format. This adjustment aims to provide patients, particularly those managing chronic conditions, with greater flexibility and time to use their prescriptions in a clinically beneficial manner. The measure was designed to eliminate the need for frequent prescription renewals, thus better aligning with patient needs and healthcare service efficiency requirements.

Medicine prescription dispensing by community pharmacists: This measure allows the delivery of medication packages for patients with chronic diseases for up to one year and facilitates digital communication between pharmacists and doctors, which is important for adherence and for reporting side effects of the drugs. The digital communication also provides access to the patient’s prescription history, which is essential for monitoring and optimizing therapeutic outcomes. In line with the objectives of the previous measure, this policy eliminates the need for chronic disease medication renewals by family doctors for up to one year, allowing patients to collect their medication every two months at a pharmacy of their choice. By decentralizing prescription renewals, the measure improves access to medications and reduces the administrative workload for family doctors and their teams. For the first time, community pharmacists in Portugal have access to medication history and the ability to send alerts or additional information to doctors through integrated digital systems.

### 3.2. Reducing Time-Consuming Processes, Streamlining Patient Management and Information Flows

NHS vaccine administration at community pharmacies without a medical prescription: The NHS expanded the administration of seasonal vaccines [flu and COVID-19] to community pharmacies without requiring a medical prescription. Vaccines are provided free of charge for patients over 60 years old, upon presentation of their citizen card, which increases accessibility and convenience. This approach has been shown to boost patient satisfaction and adherence [42]. The foundational work involved multiple partners, and an integrated information system now allows pharmacists to access vaccine history and record vaccinations for the first time in Portugal’s history.

Creation of Centers for Medical and Psychological Assessment [CAMPs]: The CAMPs were piloted in two Local Health Units [LHUs] to streamline the process of obtaining medical certificates for driving licenses, hunting permits, firearm licenses, and nautical sports certifications. In 10 months of operation, the CAMP at LHU Alto Minho conducted 731 consultations, while the CAMP at LHU Matosinhos conducted 4556 consultations. The average waiting time for a consultation ranged between 10 and 21 days [41].

Scheduling of Primary Care Services [PCS] appointments through SNS24: Appointments are now scheduled with a specific date, time, and place, addressing gaps in care and enhancing service efficiency. Previously, patients needed to contact their respective PCS directly or visit an emergency service, which resulted in high volumes of phone calls and in-person visits. This approach often led to slow response times and appointments not matching the patients’ clinical conditions.

Surgery vouchers’ digitalization: The digitalization of surgery vouchers has the potential to allow patients to schedule a needed surgery with greater speed and certainty given the possible avoidance of traditional mail. From 1 October 2023 until 19 April 2024, a total of 54,016 surgery vouchers were issued via email, representing approximately 41% of the total number of vouchers distributed. Previously, these vouchers were sent exclusively by postal mail, limiting flexibility and delivery speed.

Extension of the duration of sick leave in specific clinical conditions: The duration of sick leave for oncology, cardiac, and stroke patients has been extended to 90 days. Previously, the law limited the initial CIT to a maximum of 12 days, with extensions up to 30 days. The new legislation allows the initial TIC and its extensions to last up to 90 days for patients with cancer, ischemic heart disease, or those who have suffered a stroke. For post-operative patients, the maximum duration is now 60 days.

Electronic system for verifying temporary incapacity through medical boards for Social Security: Temporary Incapacity due to Disease is a benefit paid to workers suspected of having a disease, resulting in a temporary loss or reduction in their work or earning capacity. Verifying temporary incapacity for work, which aims to combat fraud, is carried out by a medical board through medical examinations to confirm the incapacity status of Social Security beneficiaries on sick leave and receiving sickness benefits. Previously, medical boards did not have electronic access to the patient’s medical information and relied on updated paper reports from medical doctors. This process led to avoidable visits to primary healthcare units and unnecessary consumption of family medicine consultations. With the implementation of this electronic system, it is estimated that up to 500,000 consultations per year, previously conducted solely to obtain clinical declarations for the medical board, will no longer be needed.

### 3.3. Next Moves Towards Bureaucracy Reduction

Various actions are currently underway across different projects and are being explored for future implementation. These initiatives include:

(a) Integrating digital records from both public and private medical diagnostic and therapeutic procedures: This integration offers convenience for patients and professionals, fosters continuity of care, and translates into cost reduction for the NHS by avoiding duplication of medical procedures and unnecessary clinical investigations. It also reduces the need for duplicate consultations caused by patients not having the necessary paper exams or reports available.
(b) Coupling mandatory screenings with the usual informatic medical system: Previously, mandatory screenings were managed using separate software from the day-to-day system, called SClínico (Serviços Partilhados do Ministério da Saúde (SPMS), Portugal), creating inefficiencies. Integration into the SClínico system allows family doctors to access screening test results in the same familiar interface, streamlining data management and minimizing the need for additional software.
(c) Establishing a SharePoint platform with the Social Security Institute: The platform facilitates the referral of patients who remain hospitalized due to a lack of social care or family support. This integration helps free up hospital beds needed for acute patients, optimizing resource allocation and reducing prolonged hospital stays.
(d) Employing Artificial Intelligence [AI] for automated triage in dermatology services: AI-powered screening addresses backlogs in dermatology services, which have a high number of pending consultations—65,025 as of February 2024—with an average waiting time of 6.9 months. This AI technology enables faster assessment of cases and prioritization of serious conditions such as neoplastic lesions [41,43,44].
(e) Teleconsultation through the SNS24 app or portal: Previously limited to physician-scheduled sessions, teleconsultation will now be available through the SNS24 for patients in nursing homes or long-term care units, reducing travel needs and supporting more flexible patient care management.
(f) Creation of a Digital Family Health Unit: A digital Family Health Unit is in the process of being piloted at LHU Amadora/Sintra, the LHU with the highest number of users [more than half a million registered users]. The new digital unit aims to provide healthcare access to users without a family team (which includes a designated family doctor, family nurse, and clinical secretary), attract new family doctors to the NHS, and offer digital-first healthcare solutions for a younger, digitally oriented population.
(g) Access to uniform scientific evidence for health professionals: A centralized acquisition of clinical decision support platforms is underway to standardize tools across all LHUs, ensuring consistent, evidence-based decision making and optimizing clinical outcomes.

## 4. Discussion

The key measures proposed by the EB-NHS, as outlined in the results, are analyzed here by comparing them point-by-point with findings from other countries, relevant literature, and the VBHC framework.

### 4.1. Reducing Overly Complex Regulations and Work Redundancy

These measures were created to reduce the workload pressure on primary care professionals and to avoid unnecessary visits of patients to primary care centers.

Self-certification of sickness up to three days, through contact with the SNS24: The introduction of self-certification for short-term sick leave through the SNS24 platform is a significant step toward reducing bureaucratic burden and optimizing healthcare resource utilization within the Portuguese NHS. By allowing patients to self-certify illness for up to three days, this measure addresses the inefficiencies of the traditional system, which required family doctors to issue CIT for every short-term absence, consuming valuable clinical time that could be redirected toward more critical consultations. This initiative not only simplifies the process for citizens but also decreases the risk of contagion in health centers by eliminating the need for physically ill patients to attend in-person appointments. Moreover, by shifting to a digital self-certification system, the measure promotes a higher level of control and monitoring of sick leave certifications, enhancing accountability and efficiency in healthcare services. This measure aligns with practices in other European countries, such as the UK and Sweden, where self-certification is valid for up to seven days, in Norway for four days, and in Switzerland for three days [45]. The outcomes observed in Portugal are consistent with findings from other European countries, which demonstrate that self-certification for short-term absences does not increase the duration of sick leaves and may even reduce the total number of sick leave days taken [45]. Such international evidence supports the sustainability and effectiveness of the measure in reducing unnecessary administrative burdens while maintaining control over short-term absences. Overall, this policy not only aligns with the principles of value-based healthcare by reducing non-value-added tasks but also serves as a model for other regions seeking to implement similar reforms. Future research could explore whether extending the self-certification period, as seen in other countries, would further optimize the balance between reducing bureaucratic overhead and maintaining healthcare quality and efficiency.

Issuance of digital medical certificates of illness in emergency public services and private and social healthcare services: The expansion of the authority to issue digital medical certificates for sick leave to a broader range of healthcare services represents a strategic shift toward reducing bureaucratic bottlenecks within the Portuguese healthcare system. By alleviating the burden on family doctors, who previously had almost exclusive responsibility for issuing these certificates, the measure allows these professionals to focus more on patient care, thus aligning with the principles of value-based healthcare. Expanding the issuance of medical certificates for sick leave to emergency services and private healthcare facilities embodies the core values of value-based healthcare and strengthens monitoring efforts to combat fraud in this area. Such a structural change highlights the importance of integrating private and public sectors in healthcare to optimize resources and reduce administrative delays, ensuring that the system becomes more patient centered. Future analysis could investigate the long-term impact of this redistribution on healthcare efficiency and the satisfaction levels of both patients and healthcare providers.

Extension of the prescription’s validity for medication and medical procedures up to 12 months: The extension of prescription validity from six to twelve months aligns with the core principles of value-based healthcare by reducing the administrative burden and supporting a more patient-centered approach. According to Sinsky et al. [2], separating prescription renewals from clinical appointment adherence is essential for optimizing the management of chronic illnesses. This theory suggests that providing a 12-month prescription for stable medications minimizes the repetition of administrative work, mitigates the risk of treatment interruptions, and avoids unnecessary lapses in medication access [2]. Additionally, by decoupling prescription renewal from clinical appointments, healthcare providers can ensure that patients have timely access to their medications without being solely dependent on in-person visits, thereby enhancing treatment adherence. This approach not only improves efficiency within healthcare delivery systems but also empowers patients to manage their own care more effectively. For patients with chronic illnesses, the burden of frequent clinical appointments for prescription renewal can impose significant logistical challenges, as they may already be coping with the complex day-to-day management of their conditions. Extending prescription validity provides these patients with much-needed flexibility, reduces the frequency of visits for administrative purposes, and allows them to focus on more comprehensive aspects of their healthcare. Furthermore, the policy promotes resource optimization, enabling doctors to prioritize clinical appointments for treatment optimization, disease monitoring, and patient education. Overall, the digital extension of prescription validity contributes to a more streamlined, patient-centric healthcare system, while promoting the broader goal of a paper-free NHS.

Medicine prescription dispensing by community pharmacists: The introduction of medicine prescription dispensing by community pharmacists represents a significant shift in chronic disease management by increasing the efficiency of medication delivery and enhancing patient outcomes. This approach not only improves therapeutic adherence but also supports medication reconciliation by pharmacists, ensuring that patients receive the right medications and dosages. Studies by Doshi et al. [46] and Sinsky and Moran [47] confirm that pharmacist-led interventions contribute to reducing medication-related errors, improving patient satisfaction, and decreasing unnecessary prescriptions, thereby reducing waste. Furthermore, this initiative allows family doctors to focus on more complex clinical cases by offloading routine prescription renewals to pharmacists. Studies have shown that clinical pharmacist involvement, especially in chronic disease co-management, is associated with better medication adherence and lower healthcare costs [48,49]. By allowing community pharmacists to co-manage chronic conditions and communicate directly with doctors, the measure promotes a more collaborative, team-based care approach. This innovation not only strengthens the role of community pharmacists in Portugal but also mirrors successful strategies implemented in other healthcare systems worldwide, where pharmacist involvement has been linked to improved outcomes and cost savings. Consequently, expanding pharmacist-led chronic disease management has the potential to further align healthcare delivery with value-based care principles by ensuring medication adherence, minimizing drug-related complications, and optimizing healthcare resource utilization.

### 4.2. Reducing Time-Consuming Processes, Streamlining Patient Management and Information Flows

These measures were created to simplify the patient experience with the Portuguese NHS while conserving resources and optimizing care.

NHS vaccine administration at community pharmacies without a medical prescription: The expansion of vaccine administration to community pharmacies allows for greater accessibility and proximity for older adults, improving patient experience and reducing the need for visits to NHS facilities. The inclusion of pharmacists in vaccination administration utilizes their competencies more effectively and frees up NHS nurses’ time for other essential tasks. Additionally, the implementation of an information system component enables better coordination and documentation of vaccinations, representing a significant advancement in Portugal’s healthcare service delivery.

Creation of Centers for Medical and Psychological Assessment [CAMPs]: The creation of CAMPs aims to promote greater equity in access, faster response times, and higher satisfaction levels for both patients and professionals. By removing the responsibility of issuing these certificates from the family medicine units, the initiative allows healthcare teams to focus on direct patient care, reducing the workload of family physicians. Both CAMPs view this as an opportunity to develop an independent and specialized service, enhancing the quality and capacity to monitor the process. Additionally, this separation has relieved the negative burden that these administrative tasks placed on the doctor–patient relationship [41].

Scheduling of Primary Care Services [PCS] appointments through the SNS24: The new scheduling system through SNS24 resolves many of the challenges associated with the previous process by prioritizing appointments based on clinical need rather than a first-come, first-served basis. This change not only reduces waiting times at healthcare facilities but also ensures that resources are allocated more effectively. Patients, especially those without family doctors or with urgent care needs, now benefit from more organized and accessible scheduling. Moreover, integrating emergency service appointment scheduling into the system improves access for vulnerable populations and enhances the overall patient experience by minimizing unnecessary delays and improving service coordination.

Surgery vouchers’ digitalization: The shift from postal to digital delivery of surgery vouchers enhances accessibility by offering a quicker, more efficient process that reduces costs, waste, and delivery time. Additionally, using email minimizes the risk of physical document loss and improves convenience for patients who need to schedule surgeries at alternative hospitals. By issuing 41% of the vouchers through email in just over six months, the initiative demonstrates the potential for widespread adoption of digital solutions in healthcare administrative processes, promoting a more streamlined and patient-friendly approach.

Extension of the duration of sick leave in specific clinical conditions: This extension reduces the necessity for frequent visits to family doctors, particularly for patients with limited mobility and vulnerability. By extending the validity period, the policy aims to alleviate the burden on these patients, who previously had to request sick leave at intervals that were often unsuitable for their conditions. It prevents them from having to present themselves monthly to their family doctor for bureaucratic purposes. Additionally, this approach frees up consultation periods for evaluating patients with acute illnesses, thus increasing satisfaction among both patients and family doctors.

Electronic system for verifying temporary incapacity through medical boards for Social Security: The introduction of an electronic system for verifying temporary incapacity through medical boards significantly streamlines the process by eliminating the need for patients to carry updated paper reports between doctors and the medical board. This change not only reduces the administrative burden on healthcare units but also decreases the number of unnecessary visits to family medicine consultations. As a result, it allows healthcare professionals to dedicate more time to direct patient care and reduces the strain on Primary Care Services. Additionally, the system enhances the efficiency of the medical boards’ verification process, ensuring a smoother workflow and better resource allocation within the Social Security system.

### 4.3. Next Moves Towards Bureaucracy Reduction

The measures discussed include integrating digital records, coupling mandatory screenings with the SClínico system, establishing a SharePoint platform with the Social Security Institute, employing AI for automated triage in dermatology services, implementing teleconsultation through the SNS24 app, creating a Digital Family Health Unit, and ensuring access to uniform scientific evidence for health professionals.

Integrating digital records minimizes unnecessary medical procedures and duplicate consultations, while linking mandatory screenings to the SClínico system prevents workflow disruptions caused by using separate software. This kind of digital integration is key to promoting a seamless patient experience and supporting clinical staff.

The establishment of a SharePoint platform for Social Security referrals directly addresses the issue of prolonged hospital stays due to non-medical factors, freeing up critical hospital resources and enhancing patient flow. Likewise, the adoption of AI in dermatology services tackles a major area of backlog by improving triage accuracy and reducing the burden on specialists, allowing for quicker intervention in high-priority cases.

Teleconsultation through the SNS24 app or portal expands access to care for vulnerable groups, such as residents in long-term care facilities, by eliminating the need for travel. The creation of a Digital Family Health Unit is a bold step towards transforming traditional healthcare delivery and meeting the demands of a growing population segment that prefers digital-first solutions.

Finally, ensuring uniform access to clinical decision support platforms across all LHUs is critical for reducing inconsistencies in clinical practice and improving the quality of care. This strategic approach to digital resource management is aligned with the broader objectives of the EB-NHS, promoting a more efficient, standardized, and patient-centered healthcare system.

Considering only the measures of self-certification of sickness, issuance of medical certificates for sick leave in emergency services and private healthcare services, creation of CAMPs, and the electronic system for verifying temporary incapacity through medical boards for Social Security and work purposes, it is estimated that 1.5 million consultations per year can be saved, especially in primary healthcare services. Depending on how much time an individual consultation could take, this could be equivalent to the work of about 200 to 325 family doctors if they were only performing these interventions.

### 4.4. The Impact of the Measures in Each VBHC Framework Sub-Theme

All measures were considered in the reallocation of resources from low- to high-value care, as perceived by the Expert Panel on Effective Ways of Investing in Health as the utmost necessity for sustainable and resilient European healthcare [25]. The expansion of digital medical certificate issuance upholds AV by ensuring equitable resource distribution. The extension of prescription validity for medications demonstrates AV by granting patients with chronic conditions more time for effective medication use. Both medicine prescription dispensing by community pharmacists and vaccine administration demonstrate AV by ensuring equitable access to essential medications. The creation of CAMPs aligns with AV by promoting equitable access to medical certificates, ensuring timely access regardless of geographic location. The scheduling of PCS appointments through the SNS24 ensures AV by promoting equitable access to primary care. The digitalization of surgery vouchers ensures AV by allowing patients to schedule surgery at alternative hospitals, including private facilities. The extension of sick leave duration for specific clinical conditions prioritizes AV by providing sufficient time for recovery without frequent visits to family doctors. The implementation of an electronic system for verifying temporary incapacity through medical boards prioritizes AV by transitioning from manual requests to an electronic system. Establishing a SharePoint platform with the Social Security Institute optimizes hospital resources and enhances collaboration between healthcare and social services.

TV was also contemplated on the measures below. The introduction of self-certification for sickness in Portugal optimizes TV by streamlining certificate issuance. The expansion of digital medical certificate issuance demonstrates TV by relieving family doctors and focusing on patient care. The extension of prescription validity for medications upholds TV by ensuring timely medication access. Both medicines prescriptions dispensing and vaccines administration at community pharmacies optimize outcomes through digital communication, reflecting TV. The creation of CAMPs reflects TV by streamlining the certificate-obtaining process and enabling family physicians to focus more on patient care. The scheduling of PCS appointments through the SNS24 promotes TV by organizing responses and improving healthcare system efficiency. The digitalization of surgery vouchers ensures TV by reducing costs, waste, and delivery time. The implementation of an electronic system for verifying temporary incapacity through medical boards optimizes TV by reducing unnecessary visits. The integration of digital findings from medical procedures supports TV by optimizing resource allocation. Coupling mandatory screenings with the usual informatic medical system promotes TV by streamlining data management. Employing AI for automated triage in dermatology services addresses backlogs, promoting TV.

PV highlights the enhancement of patient convenience and satisfaction, seen many times in the measures. The introduction of self-certification for sickness in Portugal honors PV by offering a convenient method for absence justification. The expansion of digital medical certificate issuance respects PV by eliminating extra appointments. The extension of prescription validity for medications respects PV by reducing the burden on patients, empowering them in self-care. Both medicines prescriptions dispensing and vaccines administration at community pharmacies show PV by empowering pharmacists and enhancing patient comfort. The creation of CAMPs supports PV by enhancing satisfaction for both patients and professionals. The scheduling of PCS appointments through the SNS24 enhances PV by improving the patient experience. The digitalization of surgery vouchers improves PV by enhancing accessibility and convenience for patients. The extension of sick leave duration for specific clinical conditions promotes PV by enhancing patient-centered care and reducing stress. The implementation of an electronic system for verifying temporary incapacity through medical boards improves PV by reducing patient burden. Teleconsultation through digital platforms supports PV by accommodating patients’ schedules. The creation of a Digital Family Health Unit enhances PV by improving accessibility, flexibility, and satisfaction. Access to uniform scientific evidence for health professionals enhances PV by improving patient safety and diagnostic accuracy.

SV, therefore, was also present in many of the changes. The introduction of self-certification for sickness in Portugal promotes SV by reducing illness transmission risks. The expansion of digital medical certificate issuance contributes to SV by promoting inclusivity and reducing barriers to healthcare access. Both medicines prescriptions dispensing and vaccines administration at community pharmacies show SV by fostering a more efficient and auditable service. The extension of sick leave duration for specific clinical conditions promotes SV by reducing stress associated with navigating healthcare bureaucracy. The implementation of an electronic system for verifying temporary incapacity through medical boards contributes to SV by enhancing access to essential services. Establishing a SharePoint platform with the Social Security Institute supports SV by enhancing collaboration. Employing AI for automated triage in dermatology services contributes to SV by enhancing equity in healthcare access. The creation of a Digital Family Health Unit supports SV by retaining and attracting specialized professionals. Access to uniform scientific evidence for health professionals supports SV by standardizing access to clinical decision support platforms.

### 4.5. Implementation Barriers and Enablers

Providing adequate support for health professionals in the NHS can minimize the bureaucratic burden associated with necessary institutionalization and administrative processes. This requires innovative technology, a strong IT infrastructure and digital literacy, an appropriately trained workforce and resources, and empowering leadership. These conditions are being achieved by integrating all levels of care into a new health structure, the LHU, that brings together primary care units and hospitals from the same region under unified leadership, budget, and goals. Furthermore, the integration of health services through LHUs can streamline communication and collaboration between different levels of care. By breaking down silos and fostering a more cohesive system, health professionals can better coordinate patient care, share information more efficiently, and avoid duplication of efforts. This not only reduces administrative burdens but also enhances the overall quality and continuity of care for patients. Moreover, the unified leadership, budget, and goals within LHUs can promote a more strategic allocation of resources and a stronger focus on population health needs. Rather than operating in isolation, primary care units and hospitals can align their priorities and work together towards common objectives, such as improving preventive care, managing chronic conditions, and addressing health disparities within their respective regions. Ultimately, this integrated approach has the potential to create a more efficient, patient-centered, and cost-effective healthcare system. By empowering health professionals with the necessary support, resources, and collaborative environment, the NHS can better meet the evolving healthcare needs of the population while reducing the administrative burdens that often hinder the delivery of high-quality care [34].

Streamlining healthcare bureaucracy in Portugal faces several barriers. Firstly, there’s a notable resistance to change within healthcare systems. Healthcare professionals and policymakers may be entrenched in traditional processes, making it challenging to introduce and adopt new approaches or technologies [50,51]. Secondly, the complex regulatory environment adds another layer of difficulty. Navigating through intricate regulations while attempting to streamline bureaucracy can be time consuming. Lastly, limited resources, including financial constraints, pose a significant barrier. Without sufficient funding, it becomes challenging to invest in the necessary infrastructure or support systems required for effective bureaucratic simplification.

Despite these challenges, several factors can aid in streamlining healthcare bureaucracy in Portugal. Firstly, strong leadership support from the EB-NHS can provide direction and momentum for reform efforts. Clear commitment and prioritization of administrative simplification at the highest levels of government can overcome resistance and drive implementation. Secondly, technological innovation presents a significant opportunity. Investments in digital infrastructure, such as electronic health records and telemedicine platforms, can automate administrative tasks and streamline processes, improving efficiency and reducing paperwork. Lastly, stakeholder engagement is essential. Involving healthcare professionals, administrative staff, patients, and policymakers in the design and implementation of new processes fosters collaboration and support for change, ensuring that reforms meet the needs of all stakeholders and are effectively implemented.

## 5. Conclusions

Delivering a strategy to integrate the European principles of value-based healthcare is a challenge for health systems and, in particular, NHS-based systems. We concluded that bureaucracy is a source of inefficiency and waste that affects all perspectives of VBHC delivery. As such, all measures to reduce bureaucracy ended up having an estimated impact on at least two of the four pillars of the VBHC framework. In fact, most measures address more than two of the pillars, with five of them estimated to address all of them. Thus, we conclude that the reduction in bureaucracy is an endeavor that will tend to address several of the pillars of the VBHC framework and should be considered as a simple method in the process of increasing VBHC. Although there is no silver bullet to address such problems, we found that several small initiatives, when strategically combined, can have a big estimated impact, releasing precious resources and contributing to the core values of VHBC. In order to maintain the move towards streamlining healthcare bureaucracy in Portugal, there will be a need to quantify the reduction in costs for the NHS, patients, and society at large of these measures. This evaluation and impact assessment represent a new phase of the project, aimed at determining the tangible benefits of the implemented measures.

During 2023 and 2024, the EB of the Portuguese NHS presented the biggest coordinated and strategic approach to fight bureaucracy in the public sector of healthcare since the creation of the Portuguese NHS in 1979. Building on the very promising impacts of the initiatives discussed in this manuscript, championed by the Portuguese NHS executive board during 2023 and 2024, actual and future decision-makers must not lose this bureaucracy fight drive. That will be one of the keys to multiplying the healthcare value delivered to patients in the years to come.

## Figures and Tables

**Table 1:** Summary of Bureaucratic Burden Impacts and Their Conceptual Framework.

**Impacts of Bureaucratic Burden**	**Concept**
Regulatory Compliance	Healthcare providers must follow numerous regulations related to patient privacy, healthcare delivery, and safety standards. Compliance requires significant documentation and constant monitoring to avoid penalties.
Documentation Requirements	Extensive medical documentation is required for continuity of care, legal protection, and payment models. EHR systems, while helpful, can also be time-consuming due to complex interfaces.
Quality Assurance and Reporting	Healthcare providers must collect, analyze, and report data to regulatory bodies to demonstrate compliance with quality standards, adding to the administrative burden.
Increased Costs	Administrative costs account for a significant portion of healthcare hours and, therefore, expenditures. They require that staff manage paperwork billing, compliance, and technology systems.
Physician Burnout	Excessive bureaucracy can lead to physician burnout as clinicians spend more time on paperwork and less on patient care, affecting both their job satisfaction and patient outcomes.
Inefficiency and Delays	Administrative complexities and delays, such as insurance approvals, can result in inefficiencies in healthcare delivery, delaying treatment and potentially compromising patient care.
**Addressing the Bureaucratic** **Burden**	**Concept**
Regulatory Reform	Simplifying and streamlining healthcare regulations can reduce unnecessary complexity, allowing healthcare providers to focus more on patient care.
Technology Solutions	Integrating more intuitive and efficient Electronic Health Record [EHR] systems and automating administrative tasks reduce the time healthcare staff spend on paperwork.
Process Improvement	Lean management techniques and other process improvements can make healthcare organizations more efficient and patient centered, reducing redundant administrative tasks.
Patient-Centered Care	Shifting the focus to outcomes that matter most to patients leads to more effective, personalized treatments and enhances patient care.
Effectiveness for Efficiency Gains	Value-based healthcare (VBHC) encourages the efficient use of resources by discouraging unnecessary procedures altogether, even if they are ‘efficient’, while prioritizing essential interventions.
Innovation Stimulus	Focus on patient outcomes can drive innovation in medical technologies and pharmaceuticals as well as healthcare delivery design, thus promoting new and better solutions.

**Table 2:** Streamlining measures implemented in the NHS distributed by the VBHC pillars, defined by the Expert Panel on Effective Ways of Investing in Health by the European Commission.

Measure	Allocative Value	Technical Value	Personal Value	Societal Value
Self-certification of sickness		✔	✔	✔
Issuance of medical certificates for sick leave in emergency services and private healthcare services	✔	✔	✔	✔
Extension of the prescription’s validity for medication and medical procedures up to 12 months	✔	✔	✔	
Medicine prescription dispensing by community pharmacists	✔	✔	✔	✔
NHS vaccine administration at community pharmacies without a medical prescription	✔	✔	✔	
Creation of Centers for Medical and Psychological Assessment (CAMPs)	✔	✔	✔	✔
Scheduling of Primary Care Services (PCS) appointments through the Portuguese NHS line (SNS24)	✔	✔	✔	
Surgery vouchers’ digitalization	✔	✔	✔	
Extension of the duration of sick leave in specific clinical conditions	✔	✔	✔	✔
Electronic system for verifying temporary incapacity through medical boards for Social Security and work purposes	✔	✔	✔	✔
Integrating findings from both public and private medical diagnostic and therapeutic procedures	✔	✔		
Coupling mandatory screenings with the usual medical system		✔	✔	
Establishing a SharePoint platform with the Social Security Institute for seamless referral to Social Security vacancies	✔			✔
Employing Artificial Intelligence (AI) for automated triage in dermatology services		✔	✔	✔
Teleconsultation through the SNS24 app or portal		✔	✔	
Creation of a Digital Family Health Unit		✔	✔	✔
Access to uniform scientific evidence for health professionals		✔	✔	

## Data Availability

Data on the issuance of self-certification of sickness were provided by SPMS—Shared Services of the Ministry of Health, with projections sourced from the EB-NHS Activities Report (Direção Executiva do Serviço Nacional de Saúde, I. P. Porto: Direção Executiva do Serviço Nacional de Saúde, I. P.; 2024. ISBN: 978-989-33-6239-6. Available from: https://www.sns.min-saude.pt/wp-content/uploads/2024/05/Relatorio_Direcao-Executiva-do-Servico-Nacional-de-Saude-IP.pdf (accessed on 24 May 2024)). Data regarding the issuance of digital medical certificates for illness in emergency public services as well as in private and social healthcare services were also supplied by SPMS. CAMP consultation data were obtained from the respective LHUs, LHU of Alto Minho and LHU of Matosinhos, that can be contacted by their respectives websites, https://www.ulsam.min-saude.pt/ (accessed on 24 May 2024) and https://www.ulsm.min-saude.pt/ (accessed on 24 May 2024), the data can also be found, partially, in the EB-NHS Activities Report (Direção Executiva do Serviço Nacional de Saúde, I. P. Porto: Direção Executiva do Serviço Nacional de Saúde, I. P.; 2024. ISBN: 978-989-33-6239-6. Available from: https://www.sns.min-saude.pt/wp-content/uploads/2024/05/Relatorio_Direcao-Executiva-do-Servico-Nacional-de-Saude-IP.pdf (accessed on 24 May 2024)) and in the EB-NHS Communiqué of 26.06.2023, available at https://www.sns.min-saude.pt/wp-content/uploads/2023/06/COMUNICADO_CAMP.pdf (accessed on 24 May 2024). Information on the digitalization of surgery vouchers was provided by SPMS. Estimates of consultations saved through the implementation of the electronic system for verifying temporary incapacity were derived from the EB-NHS Activities Report (Direção Executiva do Serviço Nacional de Saúde, I. P. Porto: Direção Executiva do Serviço Nacional de Saúde, I. P.; 2024. ISBN: 978-989-33-6239-6. Available from: https://www.sns.min-saude.pt/wp-content/uploads/2024/05/Relatorio_Direcao-Executiva-do-Servico-Nacional-de-Saude-IP.pdf (accessed on 24 May 2024)). Data on the number of pending dermatology consultations and their average waiting times were similarly extracted from the EB-NHS Activities Report Direção Executiva do Serviço Nacional de Saúde, I. P. Porto: Direção Executiva do Serviço Nacional de Saúde, I. P.; 2024. ISBN: 978-989-33-6239-6. Available from: https://www.sns.min-saude.pt/wp-content/uploads/2024/05/Relatorio_Direcao-Executiva-do-Servico-Nacional-de-Saude-IP.pdf (accessed on 24 May 2024)). The data used in this study are subject to restricted access due to legal regulations. Requests to access these data should be directed to the data owners in accordance with applicable policies and permissions.

## Abbreviations

The following abbreviations are used in this manuscript:

AV	Allocative Value
CAMP	Center for Medical and Psychological Assessment
CIT	Certificate of Temporary Incapacity
EHR	Electronic health record
EB	Executive board
EB-NHS	Executive Board of the Portuguese National Health Service
GDPR	General Data Protection Regulation
LHU	Local Health Unit
NHS	National Health Service
PV	Personal Value
SNS24	Portuguese NHS Contact Center
PCS	Primary Care Services
SV	Societal Value
TV	Technical Value
UK	United Kingdom
UN	United Nations
USA	United States of America
VBHC	Value-based healthcare
